# *In Vitro* and *In Vivo* Effects of Synthesis Novel Phenoxyacetamide Derivatives as Potent Apoptotic Inducer against HepG2 Cells through PARP-1 Inhibition

**DOI:** 10.3390/ph16111524

**Published:** 2023-10-26

**Authors:** Mai M. Sayed, Zohour I. Nabil, Nahla S. El-Shenawy, Rasha A. Al-Eisa, Mohamed S. Nafie

**Affiliations:** 1 Zoology Department, Faculty of Science, Suez Canal University, Ismailia 41522, Egypt; zoologist.mai@gmail.com (M.M.S.); zohournabil@hotmail.com (Z.I.N.); 2 Department of Biology, College of Sciences, Taif University, Taif 21944, Saudi Arabia; r.hasan@tu.edu.sa; 3 Chemistry Department, Faculty of Science, Suez Canal University, Ismailia 41522, Egypt; mohamed_nafie@science.suez.edu.eg

**Keywords:** phenoxy acetamide, HepG2, apoptosis, PARP-1, SEC-bearing model

## Abstract

To discover potential cytotoxic agents, new semi-synthetic phenoxy acetamide derivatives, compound I and compound II, were synthesized, characterized, and screened for their cytotoxic activity against breast cancer (MCF-7) and liver cancer (HepG2) cell lines. The two compounds were more promising against HepG2 than the MCF-7 cell line according to IC_50_ values. When tested against the HepG2 cell line, compound I, and compound II both had significantly increased cytotoxic activity when compared to the reference medication 5-Fluorouracil (5-FU), with IC_50_ values of 1.43 M, 5.32 M, and 6.52 M for compound 1, 5-FU and compound II, respectively. Also, compound I displayed a degree of selectivity towards cancer cells compared to normal cells. Compound I significantly enhanced HepG2 total apoptotic cell death by about a 24.51-fold increase. According to cell cycle analysis, compound I induced the arrest of the cell cycle phases G1/S and blocked the progression of the HepG2 cells. Applying the RT-PCR technique achieved a highly significant upregulation in pro-apoptotic genes. The anti-apoptotic gene was significantly downregulated. There was an intrinsic and extrinsic pathway, but the intrinsic pathway was the dominant one. Tumor growth suppression as measured by tumor weight and volume and other hematological, biochemical, and histopathological analyses confirmed the efficacy of compound I as an anticancer agent *in vivo* examination. Finally, the molecular docking study revealed that compound I was properly docked inside the binding site of PARP-1 protein with stable binding energies and interactive binding modes. Therefore, compound I shows promise as a selective anti-cancer derivative for the treatment of liver cancer after more investigations and clinical studies. This selectivity is a favorable characteristic in the developing cytotoxic agents for cancer treatment, as it indicates a potential for reduced harm to health tissues.

## 1. Introduction

Hepatocellular carcinoma (HCC), also known as hepatoma, develops from hepatocytes, the major cell type in the liver. It’s responsible for over 85% of all cases of liver cancer [1]. The majority of liver cancer diagnoses and fatalities are caused by the most common histologic type of liver cancer, known as HCC [2].

Among the available treatments are various procedures like partial liver resection and liver transplantation. However, only a few situations are thought to be appropriate for such applications. Transarterial chemoembolization, percutaneous ethanol injection, and other signal inhibitors like sorafenib [3]. Improved cancer outcomes are possible through the use of novel medications and tumor-specific delivery of FDA-approved anticancer treatments [4]. Some of the derivatives [indole-3-pyrazole-5-carboxamide analogues (10–29)] had anticancer effects against cancer cell lines that were comparable to or superior to those of sorafenib. With IC_50_ values ranging from 0.6 to 2.9 M, compound 18 demonstrated strong activity against the HCC cell lines [5].

Carboxamide acids, of which acetamide is a member, are chemical molecules having the formula CH_3_CONH_2_. Other members of this family include ethanamide and acetic acid amide. As a monocarboxylic acid amide, it belongs to the family of acetamides formed when acetic acid is condensed with ammonia [6]. It’s a solid that dissolves in water and has a musky odor and taste; it’s an incredibly weak acid found in red beetroot. Many different types of biological activity have been attributed to molecules whose central structures contain an acetamide linkage or a derivative of this connection [7]. Because of their potential therapeutic value, acetamide medicines have also received considerable attention. There is significant promise for the development of biological activities, such as anti-cancer drugs, in acetamide derivatives and their analogs [8].

The 3-(4,5-dimethylthiazol-2-yl)-2,5-diphenyl-2*H*-tetrazolium bromide (MTT) assay was used to evaluate a range of freshly discovered and synthesized acetamide sulphonyl analogs for *in vitro* cytotoxic activity against several human cancer cell lines, including HCT-1, HT-15, SF268, MCF-7, and PC-3 cells. Most of the acetamides examined in the study demonstrated potent cytotoxicity against the intended cancer cells [8].

Phenoxyacetamide derivatives were found to possess antioxidant activity, implying that these compounds have the potential to protect biological systems from the detrimental effects of oxidative processes [9,10]. Some drug classes of the phenoxy group had been discovered. They provided a thorough list of medications currently being utilized in therapy, organized into categories based on biological activity. Medications for treating neurological disorders, as well as antiviral, antibacterial, cardiac, analgesic, and anti-leukemia drugs with a variety of biological functions. The biological activity of the chemical due to the phenoxy moiety is being shown in an increasing number of articles [6,7,9]. Most frequently, the phenoxy moiety increased the likelihood that the compound would match the target, guarantee selectivity, interact with other molecules in a -bond, or boost the ability of the oxygen ether atom to create hydrogen bonds. A novel generation of medications with the terminal phenoxy group may represent the pharmacotherapy of the future [11]. Sorafenib analogues are the first treatment option for hepatocellular carcinoma. The mechanism of action involves replacing the aryl-urea component of Sorafenib with a 1,2,3-triazole ring, which serves as a linkage between the modified phenoxy fragment. The lipophilic pocket might interact hydrophobically with the terminal phenoxy group. The Huh7 hepatocellular carcinoma cell line was most responsive to this derivative with an IC_50_ of 5.67 ± 0.57 µM. In comparison to the reference standard, Sorafenib, the newly developed derivative showed a better safety profile when investigated against the MRC-5 lung fibroblast cell line. This suggests that the modified compound has a reduced impact on normal lung fibroblast cells, indicating improved safety. The substituted phenoxy group in the compound is a crucial structural element that plays a role in the safe and selective inhibition of Huh7 cells. In simpler terms, the presence of the phenoxy group enhances the new compound’s ability to bind specifically to Huh7 cells while reducing its toxicity to normal cells [12].

Some drug classes of the phenoxy group have been discovered and are currently being utilized in therapy as anti-cancer drugs like Zanubrutinib, under the brand name Brukinsa. It is used to treat the cancers mantle cell lymphoma (MCL), Waldenstrom’s macroglobulinemia (WM), marginal zone lymphoma (MZL), and chronic lymphocytic leukaemia (CLL) [13]. Moreover, Ibrutinib is a small molecule that acts as an irreversible potent inhibitor of Burton’s tyrosine kinase. It is designated as a targeted covalent drug and presented as a promising activity in B-cell malignancies in clinical trials [14]. The FDA initially gave Ibrutinib an accelerated approval in November 2013 for the treatment of MCL. However, in April 2023, the drug’s producer revoked the accelerated authorization for ibrutinib in the US. [15]. The FDA authorized Zanubrutinib for the treatment of CLL or small lymphocytic lymphoma patients in January 2023 [16].

It was possible to obtain yet another newly synthesized pyridazine hydrazide with phenoxy acetic acid added. In studies against HepG2 hepatocellular cancer cells, it was the most effective with IC_50_ of 6.9 ± 0.7 μM, as compared to the reference standard drug 5-Fluorouracil (5-FU) with IC_50_ of 8.3 ± 1.8 μM. Additionally, this substance prevented metastatic cancer cells from migrating and invading by inhibiting metalloproteinase 2 (MMP-2) and MMP-9 activity [17]. The current study aimed to investigate anti-proliferative, and apoptotic activity as the mechanism of action of two novel phenoxy acetamide derivatives (compound I and compound II) in cancer cells *in vitro* and *in vivo* to shed new light on their potential and mechanism of action.

## 2. Results and Discussion

The present study was demonstrated to explore and highlight the *in vitro* and *in vivo* cytotoxic effect of new derivatives from the phenoxyacetic acid compound. They were synthesized and fully characterized using nuclear magnetic resonance spectroscopy (NMR), ^1^H-NMR, and ^13^C-NMR, which are the most common research techniques for determining the physical and structural groups present in the compound [18]. These two compounds (I–II) were purified using crystallography and were ready to be evaluated and investigated for their cytotoxic effect against two types of cancer cell lines (MCF-7; breast cancer) and (HepG2; liver cancer) and study the mechanism of action with more focus on the most affected cell line with the lowest IC_50_ of compounds I and II.

### 2.1. Chemistry


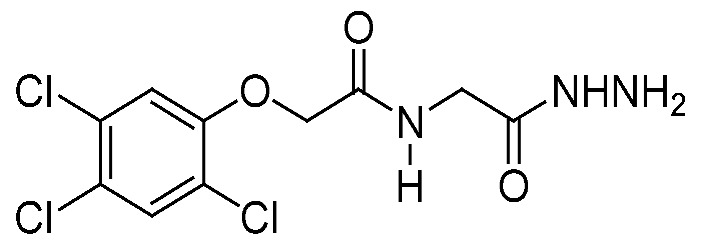


Compound I: ^1^H-NMR (DMSO) δ: 3.75 (d, 2H, *J* = 5.2 Hz, NCH_2_), 4.24 (bs, 2 H, NH_2_), 4.67 (s, 2H, OCH_2_), 7.40 (s, 1 H, Ar-H), 7.84 (s, 1 H, Ar-H), 8.20 (bs, 1 H, NH), 9.28 (bs, 1 H, NH).

^13^C-NMR (DMSO) δ: 41.0 (NCH_2_), 67.7 (OCH_2_), 126.2, 121.7, 123.7, 130.8, 131.8, 153.4 (Ar-C), 166.2, 168.2 (2 CO).


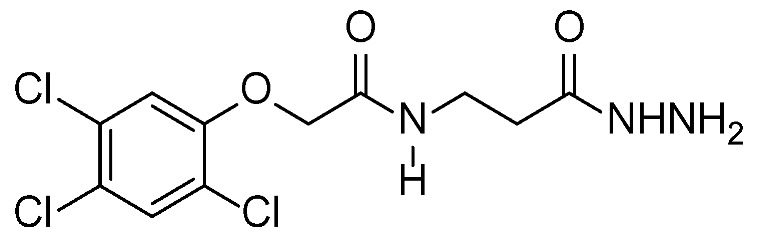


Compound II: ^1^H-NMR (DMSO) δ: 2.23–22.26 (m, 2H, CH_2_CO), 3.75 (d, 2H, J = 5.2 Hz, NCH_2_), 4.24 (bs, 2 H, NH_2_), 4.69 (s, 2H, OCH_2_), 7.40 (s, 1 H, Ar-H), 7.84 (s, 1 H, Ar-H), 8.20 (bs, 1 H, NH), 9.28 (bs, 1 H, NH).

^13^C-NMR (DMSO) δ: 33.6 (CH_2_), 35.6 (NCH_2_), 68.4 (OCH_2_), 126.1, 121.8, 123.8, 130.8, 131.1, 153.5 (Ar-C), 166.9 170.2 (2 CO) (Figures S1 and S2).

### 2.2. Cytotoxicity against Different Cell Lines

Compounds I and II exhibited a significant cytotoxic efficacy on the tumorigenic investigated cells (MCF-7 and HepG2) comparatively to the negative (un-treated cells) control and showed more potency on HepG2 cell line than MCF-7 according to the determined values of IC_50_ for each cell line (Table 1). Compound I showed more potency on the HepG2 cell line with IC_50_ of 1.43 μM than compound II with IC_50_ of 6.52 μM. Compound I had a lower cytotoxic effect on the normal/non-tumorigenic liver cells (THLE-2) with IC_50_ of 36.27 μM. The lowest determination of the IC_50_ value of compound I in all evaluated cell lines was found in the liver cancer cell line HepG2, while the highest value of IC_50_ was found in the normal/non-tumorigenic liver cells (THLE-2). It appears that the results align with a previous study conducted on novel 2-(phenoxymethyl)-5-phenyl-1,3,4-oxadiazole derivatives, which also assessed their activity toward MCF-7 cells. The substance showed notable action against MCF-7 cells in an earlier investigation [19], with an IC_50_ value of 10.51 M.

Compound I exhibited an impressive cytotoxic effect on the HepG2 cell line with IC_50_ of 1.43 μM which is lower than the IC_50_ value of 5-FU and compound II (5.32 and 6.52 μM), respectively (Table 2). In a previous study, a synthesized pyridazine hydrazide appended phenoxy acetic acid compound exhibited the highest activity against HepG2 with IC_50_ of 6.9 μM, as compared to 5-FU with IC_50_ of 8.3 μM [20]. This comparison can be relevant when considering the potential of compounds like our compound I in future research or clinical applications. It suggests that compound I may have promising cytotoxic or antitumor properties, potentially making it a valuable candidate for further investigation in the context of liver cancer treatment.

Therefore, compound I proved the remarkable ability to suppress cell viability and proliferation in a significant dose-dependent inhibition in both cancerous cell types with different IC_50_ values which were about 7.43 μM in the MCF-7 cell line and about 1.43 μM in the HepG2 cell line (Table 3). By comparing compound I cytotoxic effect on the tumorigenic MCF-7 and HepG2 cell lines based on their IC_50_ values, compound I was more cytotoxic on HepG2 cells with low IC_50_ value (1.43 µM) than MCF-7 cells with high IC_50_ value (7.43 µM). The present data represent the 5.19-fold increase in the IC_50_ value of MCF-7 cells to HepG2 cells. These results in consistent with many previous studies that confirmed the ability of anticancer agents bearing a phenoxy group to induce cell viability inhibition in different cell lines in dosage dependency [12,19].

Furthermore, the remarkable ability of compound I to suppress cell viability and proliferation in significant dose-dependent inhibition of the HepG2 cell line with IC_50_ value (1.43 µM). Also, it exhibits a mild to weak cytotoxicity against non-tumor liver cells (THLE-2) with IC_50_ value (36.27 µM), which represents a 25.36-fold increase in the IC_50_ value of THLE-2 than HepG2 cell line. These results can suggest peptide selectivity anti-proliferative action of compound I against hepatic carcinoma cells rather than normal liver cells. The recent findings showed that compound I had the strongest selective action against HepG2 cells. These results are inconsistent with a previous study of two phenoxy acetamide derivatives with selective anti-proliferative action against two different breast cancer cell lines (MCF-7 and MDA-MB-231) rather than the normal breast cell line (MCF-10A) [21]. More mechanistic investigations were conducted to track and analyze the precise method of action after the assessment derivative, compound I, demonstrated cytotoxicity against liver cancer HepG2 cells.

### 2.3. Apoptotic Evaluation

**Figure 1:**
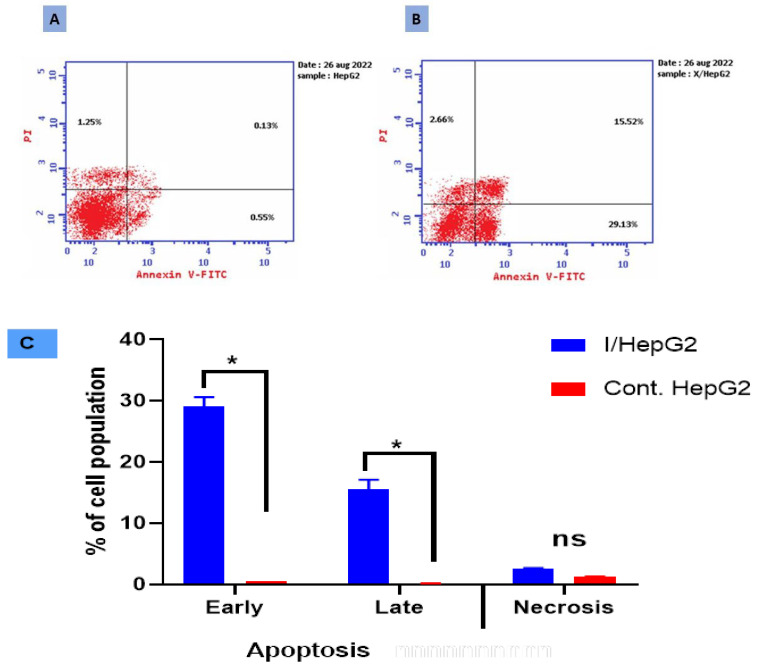
Cryptographs of annexin V/PI staining of (**A**) control (untreated) HepG2 cell, (**B**) treated HepG2 cell with compound I (IC_50_ = 1.43 µM, 48 h), displaying viable cells percentages: Q3 (normal cells, AV^−^/PI^−^), early apoptotic cells: Q4 (AV^+^/PI^−^), late apoptotic cells: Q2 (AV^−^/PI^+^), and necrotic cells: Q1 (AV^−^/PI^+^), and (**C**) Bar-representation of the percentage of cell population in the early, late apoptotic and necrotic cell. All values were expressed as mean ± SD (n = 3), and data were statistically analyzed by using an unpaired *t*-test. * Significant difference between the control and treated groups at *p* < 0.05.

HepG2 cancer cells were treated with compound I at an IC_50_ concentration of 1.43 M for 48 h to thoroughly understand the role of compound I in cell death. This was done using flowcytometric detection of counter-stained (Annexin V-FITC/PI) for detecting its apoptotic and necrotic effect and evaluating the alteration in cell cycle progression in different cell cycle phases (Figure 1). More mechanistic investigations were conducted to track and analyze the precise method of action after the investigated derivative, chemical I, demonstrated cytotoxicity against liver cancer HepG2 cells. It was found that treated cells with a concentration of IC_50_ significantly accumulated at both early (A^+^/PI^−^) and late (A^+^/PI^+^) apoptotic phases but detected cells at the early stage were relatively more than the late stage.

Compound I significantly enhanced HepG2 total apoptotic cell death by about a 24.51-fold increase (47.31% for the treated I-HepG2 cells compared to 1.93% for the untreated HepG2 control cells). In contrast to the untreated control cells’ averages of 0.55% and 0.13%, respectively, it increased the population of cells in early apoptosis to an average of 29.13% and late apoptosis to an average of 15.52% in the treated I-HepG2 cells. Additionally, this phenoxy acetamide derivative caused cell death via necrosis by inducing about a 2.12-fold increase in the necrotic cell population (2.66%) compared to negative control cells (1.25%). This suggests that compound I significantly caused apoptosis and necrosis in HepG2 cells. Comparing treated HepG2 cells to untreated control cells, there was a significant increase in the percentages of early and late apoptotic cells. In this study, it is for the first time to demonstrate, to the current knowledge, that compound I inhibits the HepG2 cells.

Relying on the literature, the late-apoptotic stage is also known as secondary necrosis, but it is different from real necrosis, and mainly characterized by involving plasma membrane permeabilization. On the other hand, the early apoptotic cell is negatively PI and positively Annexin V stained as proof that the cell membrane remains intact while exposing phosphatidylserine (PS) on the outer leaflet of the membrane, while the late apoptotic cell is positively PI and positively-Annexin V stained as a proof of loss of cell membrane integrity as well as PS externalization [22,23,24]. According to the results, compound I induces both early and late apoptosis (Figure 1), hence to verify the apoptotic stage caspases assays as well as nuclear staining are necessary, consistently [25].

In this concern, similarly, compound 16, an acetamide derivative, displaying anticancer properties against the HepG2 cell line by inhibiting cell proliferation in a dose-dependent manner and inducing the apoptotic pathway is consistent with our findings regarding the anticancer properties of compound I against HepG2 cells. Compared to HepG2 cells used as a control, it increased apoptosis rates by roughly 9-fold, both early and late. It also increased the ratio of late apoptosis from 0.49% to 5.53%, while it reduced the ratio of early apoptosis from 0.57% to 7.55%. Relative to the control, compound 16 almost increased both early and late cellular apoptosis by a factor of up to 13 [26].

**Figure 2:**
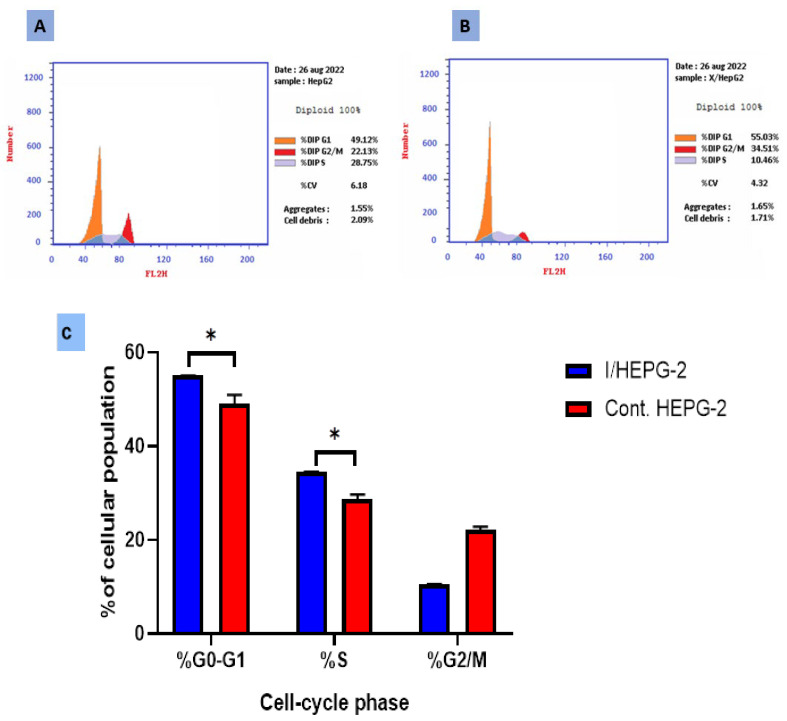
Histogram cell cycle analysis of (**A**) untreated HepG2 cells, (**B**) treated HepG2 cells (IC_50_ = 1.43 M, 48 h), and (**C**) Bar-represented cell population percentage at each cell cycle was made possible by DNA content-flow cytometry. The entire dataset was reported as mean ± SD (n = 3). * Using an unpaired *t*-test, the difference from the control group was significant at *p* < 0.05.

To summarize, DNA replication and cell division are the last steps in a sequence of developmental events known as the cell cycle (Figure 2) that may be divided into four distinct phases: G1, S (synthesis), G2, and M [27]. Compound I was chosen for further examination based on the promising results from the MTT experiment to assess its influence on the apoptotic process and investigate its capacity to play an active role in cell cycle progression in HepG2 cells. Also, the proportion of cells in each proliferation phase after treatment with compound I was calculated using cell cycle analysis.

The percentage of cells in the G1 and S phases was also considerably higher in all evaluated cells compared to untreated control HepG2 cells. Cells treated with compound I exhibited distinct changes in their cell cycle and apoptosis compared to untreated control cells. The treated cells displayed an increase in DNA content at both the G0/G1 phase (55.03% compared to 49.12% in control cells) and the S phase (34.51% compared to 28.75% in control HepG2 cells). This suggests that the compound caused cell growth arrest at these two phases of the cell cycle. The treated cells exhibited characteristics associated with apoptosis. This was indicated by an increase in annexin V-positive/PI-negative staining, which is often observed in the early stages of apoptosis. Moreover, there was an increase in double-positive staining cells, which typically represent cells in late apoptosis. These findings suggest that compound I induced both early and late apoptosis in the treated cells. Compound I caused the cell cycle phases G1/S to be arrested and stopped the proliferation of HepG2 cells. However, the treatment decreased cellular populations at the G2/M phase (10.46% compared to 22.13% for the untreated cells). These findings are in agreement with those of prior research that found that a new 2-amino benzamide derivative had the strongest activity against the HepG2 hepatocellular cancer cell line (IC_50_ of 3.84 ± 0.54 μM). The anticancer mechanism may have involved cell cycle arrest at the G2/M phase and apoptosis [28].

### 2.4. Pro-Apoptotic and Anti-Apoptotic Genes Using RT-PCR

**Figure 3:**
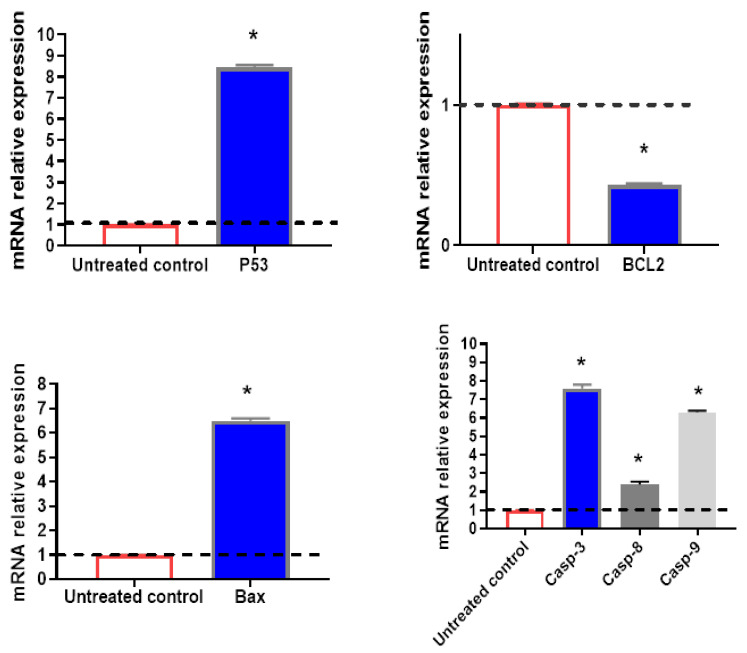
The mRNA relative expression (presented as fold change) of selected significantly regulated genes. Values above the black line are upregulated, while values below the line are downregulated. All values were expressed as mean ± SD (n = 3). * Significantly (*p* < 0.05) different from the untreated control group using an unpaired *t*-test.

The apoptosis-inducing activity of the tested compound I in HepG2 cells was further validated by examining the expression levels of genes involved in apoptosis in both untreated and treated HepG2 cells using RT-PCR (Figure 3). Compound I achieved a highly significant upregulation, compared to the control group, in pro-apoptotic genes, the treatment increased the *p53* level by 8.45-fold, *Bax* gene level by 6.5-fold, *casp-3* level by 7.6-fold, *casp-9* by 6.3-fold, respectively, it also achieved a significant upregulation in *casp-8* with 2.4-fold. However, the anti-apoptotic gene, the *BCL2* gene was significantly downregulated by 0.43-fold. There was an intrinsic and extrinsic pathway in the apoptosis process, but the intrinsic pathway is the dominant one. These findings are consistent with the flow cytometric evidence linking compound I dependence and apoptotic activation in HepG2 cells.

Additionally, the present study agrees with another previous study, in which sulfonamide containing nimesulide derivatives possessed the most effective compounds against HT-29 colon cancer and the MCF-7 breast cancer cell line with IC_50_ of 9.24 μM and 11.35 μM, respectively [29]. BCL-2-associated X protein (*Bax*) upregulation and B-cell lymphoma 2 (*BCL-2*) downregulation were shown to be the underlying mechanisms of the anticancer effect [30].

Both death receptor-mediated (or extrinsic) and mitochondria-dependent mechanisms play significant roles in apoptosis (or intrinsic). The activation of the former route begins with the binding of a ligand to a death receptor. To induce apoptosis, the primary death receptor (Fas) and associated ligand is tumor necrosis factor-alpha (TNF-α) (TRAIL). When activated by Fas ligand (FasL) or TRAIL, respectively, death receptors DR4 and DR5 bind procaspase 8 and trigger cell death. *Casp-8* is activated because of this process. The latter causes apoptosis by either directly activating casp-3 or by cleaving BID (BH3 interacting domain death agonist), both of which cause mitochondrial dysfunction, the release of cytochrome C, and the activation of *casp*-9 and -3. Apoptosis is characterized by DNA fragmentation and cell death, both of which are facilitated by *casp-3* [31].

Members of the BCL family that are attached to the mitochondrial membrane have an impact on the mitochondrial pathway. *Bax* and *BCL-2* are examples of these proteins, which are either pro- or anti-apoptotic regulatory proteins. The proapoptotic proteins BCL-2 associated X protein (Bax), BCL-2 homologous antagonist/killer (Bak), and BID enhance cytochrome c release from mitochondria whereas the antiapoptotic proteins BCL-2 and Bcl-XL prevent it. The apoptosis-mediating executioner protease procaspase 9 is drawn to and activated by cytochrome C and deoxyadenosine triphosphate (dATP), which in turn activates *casp-3* and causes cell death. Apoptotic protease activating factor (APAF-1) is then recruited by this multimeric complex [32,33].

Based on the present study, compound I cytotoxic efficacy can be attributed to exerting dual programmed cell death mechanisms at the same time, one associated with the highly significant upregulation in the pro-apoptotic genes, *p53*, *Bax*, *casp-3*, *casp-8*, and *casp-9* level, as the most important evidence of intrinsic-apoptotic-cascade pathway and the other one associated to the significant downregulation in the *BCL-2* gene which is considered as an anti-apoptotic gene and its primary function is to suppress pro-apoptotic signals, which allows the cancer cell to survive in challenging circumstances. So, when compound I decrease the *BCL-2* level it helps the cell undergo apoptosis, consistent with the previous investigations [34,35,36].

Therefore, the novel findings about the efficacy of compound I on human liver cancer cell line as an anti-proliferative, cell cycle blocker in critical phases (G1/S phase) and activator of programmed cell death via intrinsic and extrinsic apoptotic cascade pathway render it to be a promising modality in more specific and effective anti-liver cancer drugs.

### 2.5. In Vivo Results

#### 2.5.1. Antitumor Assays

Measurements of tumor growth (tumor weight and volume) and hematological, biochemical, and histological analyses were performed to determine the efficacy of compound I as an anticancer agent in an *in vivo* experiment. The lethal dose of compound I was first trialed in a preliminary experiment. The LD_50_ of compound I was demonstrated to be 40 mg/kg B·wt. Compound I’s anti-proliferative activity was evaluated in SEC-bearing mice and compared to that of conventional treatment (5-FU) and positive control (SEC control).

When SEC cells were implanted into mice, the tumor volume increased (Table 4); however, treatment with compound I significantly decreased tumor volume, bringing it down to about 1260 mm^3^ as opposed to 4960 mm^3^ in the SEC group. In addition, compared to 5-FU, which is only able to suppress the growth of SEC cells by around 64.51%, compound I was able to produce a substantial decrease in the tumor inhibition ratio percentage (TIR%) by 74.59%. Also, it achieved a significant reduction in tumor mass. These results are consistent with a recent study of bisamides and thiazole derivatives that confirmed their ability to increase the TIR% and suppress tumor growth [37].

#### 2.5.2. Hematological Assays

According to the hematological parameters of the normal mice treated with compound I, there were no significant changes observed in complete blood count (CBC) parameters when compared to the normal control animals. This lack of significant alteration in CBC parameters indicates that the investigated compound I did not have any adverse effects on the blood composition of the treated mice (Table 5). This finding suggests that compound I has a wide safety margin when administered to normal mice. All CBC parameters were shown to have shifted due to solid tumor proliferation when comparing the SEC control animals and the negative control group.

The present study’s findings showed that compound I treatment of SEC-bearing mice significantly increased their RBC count (6.66 × 10^6^/µL), as compared with the SEC positive control group (5.97 × 10^6^/µL). There was a significant increase in Hb of SEC-bearing mice treated with compound I (12.76 g/dL), as compared with the SEC group (10.03 g/dL). The WBC count between the SEC positive control (10.86 × 10^3^/µL) and the treated animals with compound I (9.90 × 10^3^/µL) did not significantly differ. There was a significant decrease in the lymphocytes of SEC-bearing mice treated with compound I, as compared with the SEC group (Table 5). There was a highly significant decrease in the granulocyte of SEC-bearing mice treated with compound I, as compared with the SEC-positive control group. All of these findings are in line with earlier research on innovative bis-amide-based bis-thiazoles that act as anti-colorectal cancer medications [37].

The elevation of WBC, reduction of Hb, and decrease in the number of RBCs were observed in SEC mice compared to negative control mice. This may happen due to hemolytic disorders, as previously mentioned, or iron insufficiency [38]. However, administration of compound I increased the amount of Hb, and RBCs accompanied by a decrease in WBCs amount, lymphocyte, and granulocyte percentage. The increase in WBCs, lymphocyte, and granulocyte percentage within the SEC control group could be due to the acute inflammatory response or stress due to the proliferation of SEC cells [39]. The decrease in WBCs, lymphocyte, and granulocyte percentage within SEC-bearing mice treated with compound I might be due to the main goal of cancer treatment being to control the disease. Chemotherapy is administered to these patients to reduce the size of their tumors and/or halt the progression of their cancer [40].

According to the biochemical parameters, AST, and ALT activities as well as creatinine levels were evaluated to find out the effect of compound I in SEC-bearing mice in comparison with normal mice and untreated SEC-bearing mice (Table 5). There was no evidence of toxicity in the liver or kidneys of regularly treated mice after receiving compound I, as was the case with the hematological profile. Comparing the untreated SEC group, the ALT and AST were elevated to 62 U/L and 139 U/L, respectively compared to the normal control mice (ALT and AST 40 U/L and 71 U/L, respectively). Treatment with compound I reduced the increased ALT and AST activity to 47.66 U/L and 111 U/L, respectively, due to the spread of malignancy. These results are in line with a previous study on 4-octadecanylamino-benzoyl-alpha-phenoxy-N-(2-chlorophenyl)-acetamide as one of the active compounds in *Acacia ferruginea* methanolic extract [41]. In addition, treatment by compound I maintained the creatinine level near normal. There was no significant difference in creatinine levels between all groups. These findings contradict those of a previous study that evaluated renal indicators in an animal model of visceral leishmaniasis using quinazolinone-based acetamide derivatives. Neither urea nor creatinine levels varied in a discernible manner [42].

#### 2.5.3. Histopathological Observation

**Figure 4:**
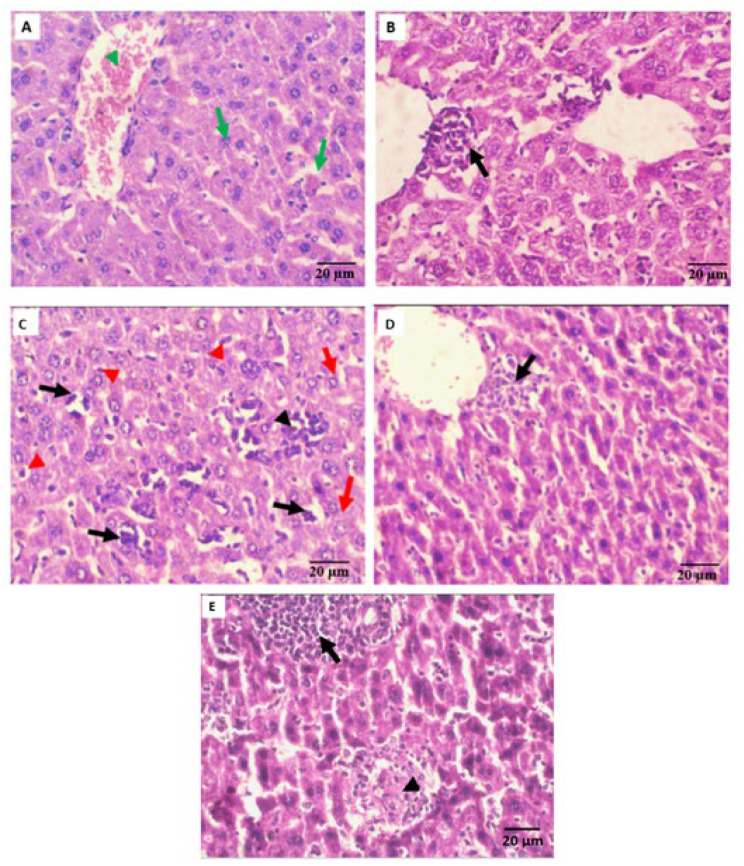
Photomicrograph showing T.S of liver of (**A**) Represents negative control mice, Uniform liver tissue showing plates of hepatocytes with patent sinusoids (Green arrows) and mildly congested vessels (Green arrowhead) (H&E, 40×), (**B**) Represents compound I treated normal mice, Liver tissue show areas of perivenular lobular inflammation (Black arrows) (H&E, 40×). (**C**) Represents positive control SEC-bearing mice, liver tissue shows foci of lobular inflammation (Black arrows). There was an area of necrosis (Black arrowhead) with the replacement of hepatocytes by chronic inflammatory cells. Many hepatocytes show hydropic degeneration (Red arrows) and scattered apoptotic cells (Red arrowhead) (H&E, 40×), (**D**) Represents 5-FU treated SEC-bearing mice Liver tissue shows fewer foci of lobular inflammation (Black arrows). No areas of necrosis. No evidence of hydropic degeneration or apoptotic hepatocytes (H&E, 40×), and (**E**) Represents compound I treated SEC-bearing mice, liver tissue shows fewer foci of lobular inflammation (Black arrows). There are areas of necrosis (Black arrowhead) with the replacement of hepatocytes by chronic inflammatory cells. No evidence of apoptosis or hydropic degeneration in hepatocytes. (H&E, 40×).

Through the histopathology of liver tissue, the hepatocytes of negative control mice showed uniform plates of hepatocytes with patent sinusoids (Figure 4). There is no change in the histological parameters. However, the liver of normal mice which are treated with compound I showed near normal hepatocytes with a peri-venular lobular inflammation. With regards to the liver of positive control SEC-bearing mice, it showed hydropic degeneration, lobular inflammation, apoptosis, and confluent necrosis. The liver of SEC-bearing mice treated with 5-FU showed no hydropic degeneration and lobular inflammation, while the liver of SEC-bearing mice treated with compound I showed lobular inflammation and confluent necrosis. The results are in parallel with a previous study on 4-(Aminomethyl)-6-(trifluoromethyl)-2-(phenoxy)pyridine derivative [43].

**Figure 5:**
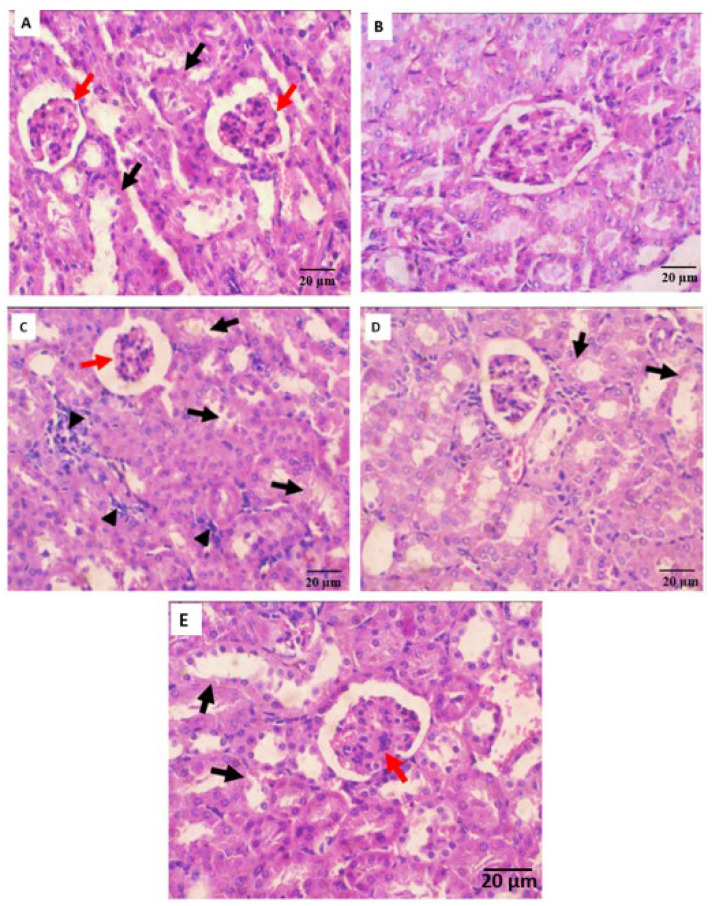
Photomicrograph showing T.S of kidney of (**A**) Represents normal control mice, renal tissue shows uniform tubules (Black arrows) Glomeruli (Red arrows) No evidence of interstitial inflammation (H&E, 40×), (**B**) Represents compound I treated normal mice, and uniform renal tissue showing no evidence of tubular or glomerular injury or chronic interstitial inflammation (H&E, 40×). (**C**) Represents positive control SEC-bearing mice, renal tissue shows evidence of acute tubular injury in many tubules (Black arrows), and foci of chronic interstitial inflammation (Black arrowheads). No evidence of any glomerular lesions (Red arrows) (H&E, 40×), (**D**) Represents 5-FU treated SEC-bearing mice, and renal tissue shows evidence of acute tubular injury in a few tubules (Black arrows). No evidence of chronic interstitial inflammation (H&E, 40×), and (**E**) Represents compound I treated SEC-bearing mice, renal tissue shows few tubules with evidence of tubular injury (Black arrows). Glomeruli show mild mesangial expansion (Red arrows). No evidence of chronic interstitial inflammation (H&E, 40×).

The kidney of negative control mice shows uniform tubules and glomeruli. There is no evidence of interstitial inflammation (Figure 5). The kidneys of normal mice treated with compound I showed renal tissue with no evidence of tubular or glomerular injury or chronic interstitial inflammation. With regards to the kidney of positive untreated SEC-bearing mice, it showed renal tissue with evidence of acute tubular injury in many tubules, 50–75% of tubules show evidence of ATI and foci of chronic interstitial inflammation. No evidence of any glomerular lesions. The kidney of SEC-bearing mice treated with 5-FU shows renal tissue with evidence of acute tubular injury in a few tubules, 25–50% of tubules show evidence of ATI with no evidence of chronic interstitial inflammation. However, the kidney of SEC-bearing mice which were treated with compound I showed renal tissue with few tubules and had no evidence of tubular injury. Glomeruli showed mild mesangial expansion without indications of persistent interstitial inflammation. These results are inconsistent with a previous study on 4-(Aminomethyl)-6-(trifluoromethyl)-2-(phenoxy)pyridine derivatives [44].

**Figure 6:**
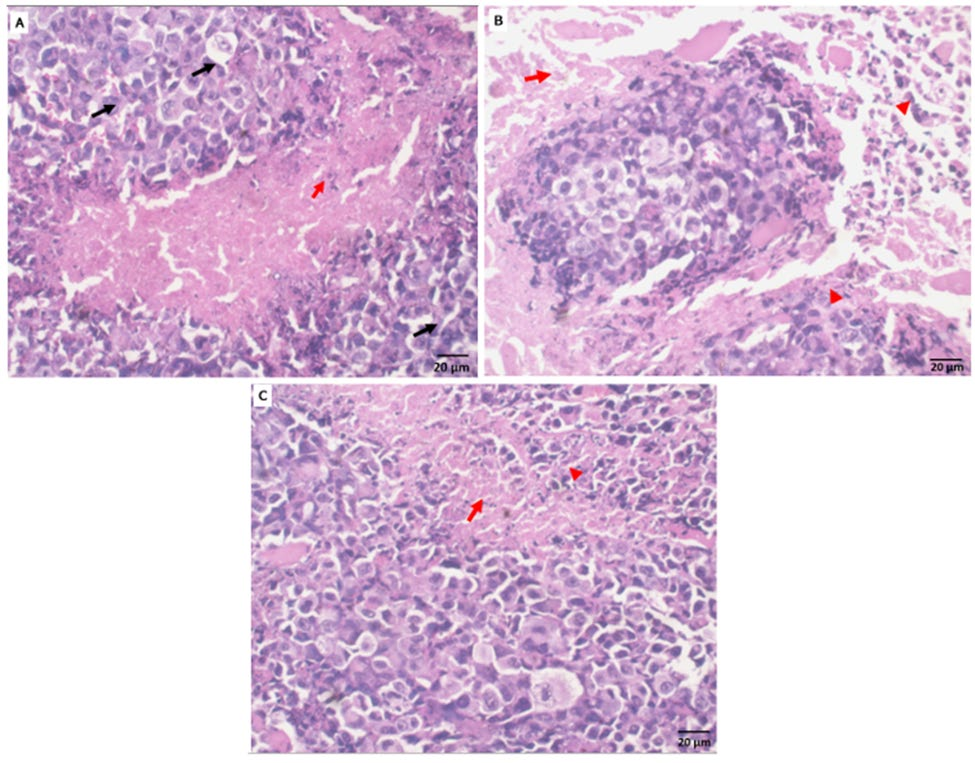
Photomicrograph showing T.S of Solid Ehrlich carcinoma of (**A**) Section of Ehrlich tumor-bearing mice, Tumor tissue is composed of groups and sheets of malignant epithelial cells (Black arrows), showing cellular and nuclear pleomorphism, nuclear hyperchromasia, and increased nucleo-cytoplasmic ratio. There are areas of tumor necrosis (Red arrows) (H&E, 40×), (**B**) Section of Ehrlich tumor-bearing mice treated with 5-FU, Tumor tissue shows multiple areas of necrosis (Red arrows) and chronic inflammation (Red arrowheads). Tumor cells show decreased cellular and nuclear pleomorphism (H&E, 40×) and (**C**) Section of Ehrlich tumor-bearing mice treated with compound I. Tumor tissue shows scattered areas of necrosis (Red arrows) and chronic inflammation (Red arrowheads). Tumor cells show decreased cellular and nuclear pleomorphism (H&E, 40×).

Histopathological analysis of tumors in mice harboring SEC reveals compact tumor tissue made up of clusters and sheets of malignant epithelial cells with features of malignancy such as cellular and nuclear pleomorphism as well as nuclear hyperchromasia, and an elevated nucleo-cytoplasmic ratio (Figure 6). Bundles of skeletal muscle are being invaded by tumor cells. No evidence of necrosis or chronic inflammatory infiltration. Tumor tissue in SEC-bearing mice treated with 5-FU demonstrates persistent inflammation and many regions of necrosis. Cellular and nuclear pleomorphism are both reduced in tumor cells. About 25% of tumor tissue in SEC-bearing mice treated with a compound I display scattered regions of necrosis and persistent inflammation. Newly synthesized Indolephenoxyacetamide (IPA) analogs consistently reduce cellular and nuclear pleomorphism in tumor cells, suggesting they inhibit tumor growth by inhibiting angiogenesis [6].

Therefore, the *in vivo* study revealed that compound I can be viewed as an effective agent by reducing the TIR% and tumor mass as committed. It decreased the elevated hematological and biochemical parameters and maintained them near normal levels in SEC-bearing mice. Regarding the histopathological examination compound, I also stopped the deterioration of cancer cells and prevented them from spreading. We can conclude that compound I can be viewed as a promising anti-cancer phenoxy acetamide derivative for the treatment of liver cancer that is somewhat selective. 

### 2.6. Molecular Docking 

**Figure 7:**
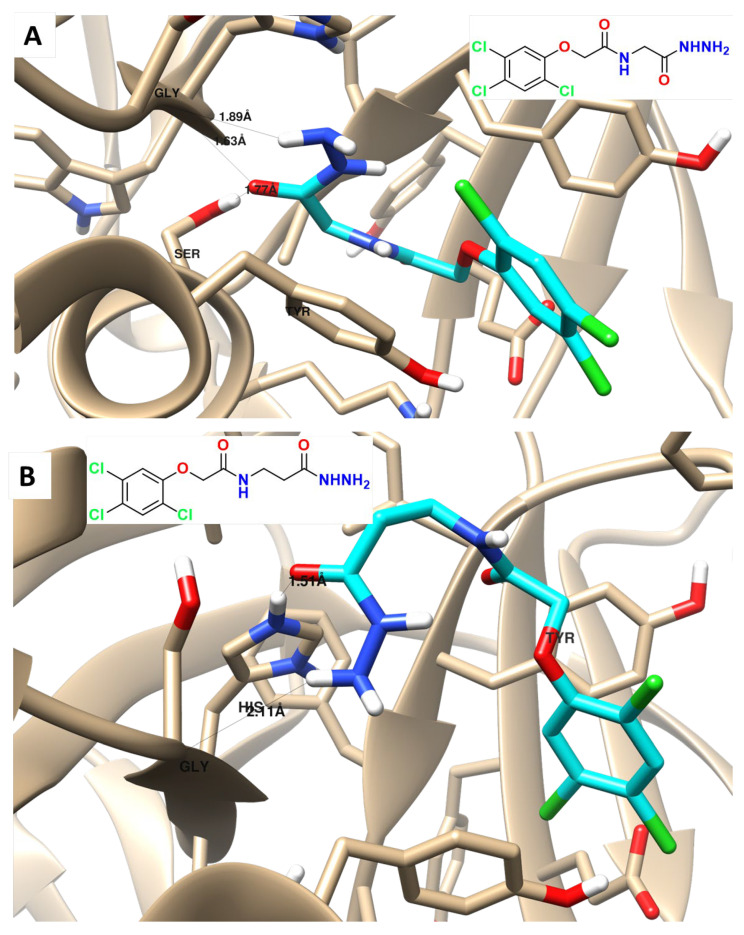
Binding disposition, and interactions of the docked compound I (**A**) and II (**B**) towards PARP-1 protein.

Finally, molecular docking was performed to illustrate how the most active compound, compound I, binds with its specific target (PARP-I) and to see the virtual mechanism of compound I and II (Figure 7) binding towards the PARP-I target protein and its binding site. The results revealed that compound I was properly docked inside the binding site of PARP-1 protein with stable binding energies and interactive binding modes. Compound I formed two H-bond interactions with Gly 863 and one H-bond with Ser 904, it formed arene-arene interactions with Tyr 896, while Compound II formed one H-bond interaction with Gly 863 and one H-bond with His 904 (Figure 7A,B). Docking results agreed with the PARP-1 inhibition assay, as an experimental approach, results revealed that compound I exhibited potent PARP-1 inhibition by 92.1% with an IC_50_ value of 1.52 nM compared to Olaparib (IC_50_ = 1.49 nM).

These data are consistent with a recent study on new bis(1,2,4-triazolo[3,4-b][1,3,4]thiadiazines) and bis((quinoxaline-2-yl)phenoxy)alkanes as dual PARP-1 and EGFR targets are inhibited by anti-breast cancer treatments [21].

## 3. Materials and Methods

### 3.1. Chemistry

#### 3.1.1. Method for Preparation of Phenoxy Acetamide Derivatives

**Scheme 1:**
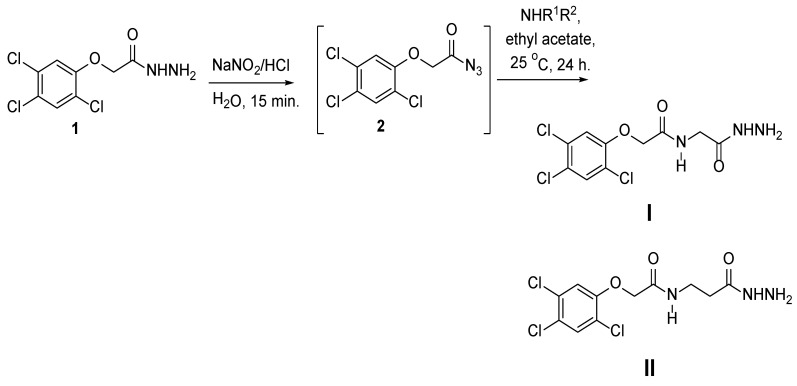
Preparation of trichlorophenoxy derivatives I and II.

The precursor 2,4,5-trichloro-phenoxyacetic acid hydrazide (1) was prepared in excellent yield by the reaction of the available methyl 2,4,5-trichloro-phenoxyacetate with hydrazine hydrate in ethanol 95% for 4 h at 78 °C (Scheme 1). 2,4,5-Trichloro-phenoxyacetic acid hydrazide (1) is an excellent precursor for phenoxy acetic acid modification by attachment of either amines or amino acid residues through a peptide bond via azide coupling method [45]. 

The solvent used in the experiment was purified and dried using standard methods, as described by Patil and Rathod [46]. “The boiling range of the petroleum ether used was 40–60 °C. Thin layer chromatography (TLC): silica gel 60 F_254_ plastic plates (E. Merck, layer thickness 0.2 mm) detected by UV absorption. Elemental analyses were performed on a Flash EA-1112 instrument at the Microanalytical Laboratory, Faculty of Science, Suez Canal University, Ismailia, Egypt”. The values for the melting points are uncorrected and were obtained using a Buchi 510 melting-point equipment. Nuclear magnetic resonance (NMR) spectra were measured using Tetramethyl silane (TMS) with a Bruker 300 MHz that was utilized as internal standards.

#### 3.1.2. Synthesis of 2,4,5-Trichloro-Phenoxyacetic Acid Hydrazide (1)

Hydrazine hydrate (5 mL, 80.0 mmol) was added to a solution of ethyl 2-(2,4,5-trichlorophenoxy) acetate (10.0 mmol) in ethanol (30 mL). The reaction mixture was reflexed for 6 h after cooling to room temperature. The precipitate was subsequently removed using a filter, washed with ice-cold ethanol and ether, and then recrystallized using ethanol. Colorless crystals yield (87%) with m.p. 218–220 °C (lit 221 °C) [47].

### 3.2. Cytotoxic Activity

The cytotoxic activity of two synthesized compounds (I and II) against different cell lines (MCF-7, HepG2, and THLE-2) using the MTT assay. A popular technique for determining cell viability and cytotoxicity is the MTT test. These cell lines were purchased from the ATCC (American Type Culture Collection), and they were cultivated using Freshney’s recommended methodology [48]. The 3-(4,5-methyl-2-thiazolyl)-2,5-diphenyl-2H-tetrazolium bromide (MTT), a yellow substance, is reduced to a purple formazan product in the MTT experiment. This reduction mainly occurs due to mitochondrial reductase activity within living cells. Serial concentrations of the compounds (I and II) were prepared for treatment. These concentrations were 100, 25, 6.30, 1.6, and 0.4 μM. The prepared compounds were added to the cultured cells, and the cells were then incubated for 48 h. After the incubation period, the percentage of cell survival was determined. This was done by measuring the optical absorbance at λ570 nm. The absorbance values at this wavelength are indicative of the amount of formazan product formed, which in turn reflects cell viability: $\mathrm{cell}\;\mathrm{viability}\;\left(\%\right)=\frac{A\;\mathrm{sample}}{A\;\mathrm{control}}$ × 100, then IC_50_ values were determined by GraphPad Prism software version 8.0.

### 3.3. Apoptotic Evaluation

The apoptotic impact of treatment with compound I on the HepG2 cell line was evaluated. This type of analysis is commonly performed in cell biology and drug development research to understand how a compound affects cell death processes. Annexin V/PI staining is a widely used method to assess apoptosis in cells. A protein called annexin V interacts with the phosphatidylserine that is visible on the outer membrane of apoptotic cells. PI (propidium iodide) is a dye that stains the DNA of cells with compromised cell membranes, such as late-stage apoptotic or necrotic cells. Flow cytometry is a technique that allows for the quantitative analysis of individual cells in a population. By staining HepG2 cells with Annexin V and PI and then analyzing them using flow cytometry, we can distinguish between live cells, early apoptotic cells (Annexin V positive, PI negative), late apoptotic cells (Annexin V positive, PI positive), and necrotic cells (Annexin V negative, PI positive). This helps assess the extent of apoptosis induced by compound I. Real-time polymerase Chain Reaction (RT-PCR) is a molecular biology technique used to evaluate gene expression levels. This comprehensive approach helps to evaluate the apoptotic impact of compound I on HepG2 cells at various levels, from cellular morphology and cell cycle progression to the molecular expression of genes involved in apoptosis. These methods help provide a deeper understanding of how the compound may be affecting cell death processes in the HepG2 cell line.

### 3.4. Annexin V/PI and Cell Cycle 

Using a flow cytometer (FACSCalibur, Becton-Dickinson, San Jose, CA, USA) and an annexin V-FITC/PI double labeling detection kit (BioVision Research Products, Waltham, MA, USA), apoptosis in cells was identified. Briefly, 1 × 10^6^ HepG2 cells were first planted in each well of a 6-well culture plate. The cells were cultured at 37 °C with 5% CO_2_ for 24 h to allow them to adhere and reach an appropriate growth phase. After the initial 24-h culture period, an IC_50_ concentration of compound I was added to the cell culture. The cells were then incubated with compound I for an additional 48 h. This extended incubation period allows for the assessment of long-term effects on cell viability and apoptosis. To prepare the cells for analysis, they were harvested by trypsinization. This process involves the use of trypsin, an enzyme that detaches adherent cells from the culture plate. After trypsinization, the cells were collected and prepared for the subsequent steps. The harvested cells were washed twice with phosphate-buffered saline (PBS) to remove any remaining culture medium or trypsin. After washing, the cells were centrifuged to form a pellet. The cell pellet was suspended in 500 μL of Annexin V binding buffer. Annexin V binding buffer is a solution optimized for the Annexin V/PI staining assay. Annexin V-FITC, a fluorochrome-conjugated protein that binds to phosphatidylserine exposed on the surface of apoptotic cells, was added to the cell suspension. Propidium iodide (PI, Sigma–Aldrich, St. Louis, MO, USA) solution was also added. PI is a dye that can penetrate the compromised membranes of late-stage apoptotic or necrotic cells and stain their DNA. The staining process followed the manufacturer’s protocol provided by BioVision Research Products. Using a FACSCalibur flow cytometer (from Becton-Dickinson), flow cytometry analysis of the stained cell suspension was then performed. The flow cytometer measures the fluorescence signals emitted by Annexin V-FITC and PI, allowing for the quantification and differentiation of life, early apoptotic, late apoptotic, and necrotic cells based on their staining patterns [2].

HepG2 cells were initially seeded in 6-well plates and cultured until they reached a cell density of 80%, indicating that they were actively growing and ready for analysis. After reaching the appropriate density, the cells were treated with an IC_50_ of compound I for 48 h. This treatment period allows for the assessment of the compound’s impact on cell cycle progression. After the 48-h treatment, 1 × 10^6^ cells were collected for cell cycle analysis. The collected cells were fixed in 70% ethanol at 4 °C overnight. Ethanol fixation helps preserve the cellular structure and DNA content for subsequent analysis. To analyze the cell cycle distribution, the fixed cells were labeled with PI for 30 min. RNase A (1% RNase A) was included during the staining process. RNase A helps to degrade RNA in the cell, leaving only DNA for analysis. PI is a fluorescent dye that binds to DNA, allowing for the quantification of DNA content in each cell. It stains DNA in a concentration-dependent manner, so cells in different phases of the cell cycle will exhibit different levels of fluorescence. Flow cytometry was performed using a flow cytometer equipped with a laser emitting at 488 nm for excitation and detecting emissions at 630 nm. The flow cytometer measures the fluorescence emitted by PI-stained DNA, allowing for the quantification of DNA content in individual cells. Based on the DNA content, cells were categorized into different phases of the cell cycle: G0/G1 (cells with diploid DNA content), S (cells with increased DNA content due to DNA replication), and G2/M (cells with diploid DNA content after DNA replication but before cell division). Data analysis was carried out using CellQuest Software (Version 5.1) from Becton-Dickinson, BD, Erembodegem, Belgium. The results were typically displayed as histograms, which show the distribution of cells in G0/G1, S, and G2/M phases based on their DNA content. 

### 3.5. Real-Time Polymerase Chain Reaction (RT-PCR)

HepG2 cells were cultivated for 24 h to allow them to adhere and enter the proper growth phase. They were planted in 6-well culture plates at a density of 2 × 10^5^ cells per well. After this initial culture period, two sets of cell cultures were prepared: a control group and a treated group. The treated group was exposed to an IC_50_ concentration of compound I for an additional 48 h. Total RNA was isolated from both the control and treated cells using Trizol reagent, a widely used reagent for RNA extraction. This step is crucial for obtaining high-quality RNA for subsequent RT-PCR analysis. To evaluate the expression of particular genes, RT-PCR was used. SYBR Green PCR Master Mix from BioRad was used for the RT-PCR reactions. Using SYBR Green, a fluorescent dye that binds to double-stranded DNA, it is possible to track the PCR product’s amplification in real-time. The RT-PCR reaction was set up according to the manufacturer’s instructions for the BioRad SYBR Green PCR Master Mix [49].

PCR primers for amplifying specific gene targets (e.g., *P53*, *Bax*, *BCL2*, *Casp-3*, *Casp-8*, and *Casp-9*) were designed using Rotor-Gene RT-PCR Software 1.7 from Corbett Research (Table 6). The primers are crucial for specifically amplifying the target gene sequences during the PCR reaction. The RT-PCR machine was used to amplify the target gene sequences. SYBR Green fluorescence levels were monitored in real-time as the DNA amplified. The cycle thresholds (Ct) were recorded, which indicate the cycle number at which the fluorescence signal surpasses a certain threshold, reflecting the amount of amplified DNA. The relative gene expression levels were calculated using the ΔΔCt method. This method compares the Ct values of the target genes (*P53*, *Bax*, *BCL2*, *Casp-3*, *Casp-8*, and *Casp-9*) to reference or control genes and normalizes them to the untreated control group. The results were typically presented as the mean ± standard deviation (SD) of the mean from triplicate experiments.

### 3.6. In Vivo Anti-Tumor Study

**Figure 8:**
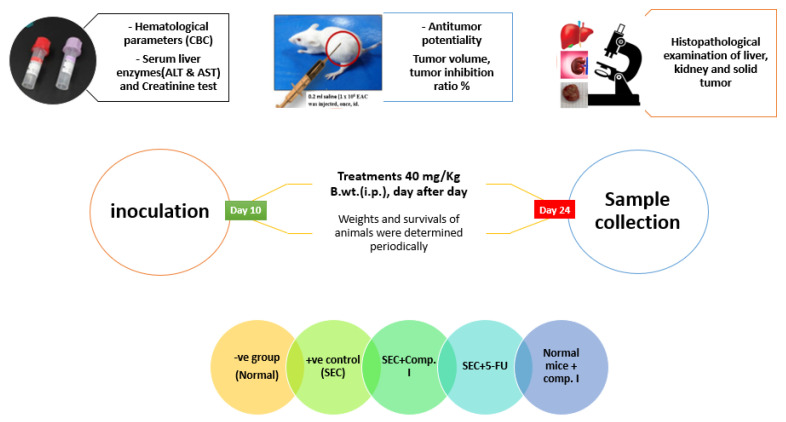
Summary of *in vivo* experiment workflow.

The purpose of Figure 8 is to provide an overview of the study’s design and methods across different aspects, highlighting how we investigated the antitumor potential of compound I while also assessing its effects on hematological, biochemical, and histopathological parameters [50,51].

#### 3.6.1. Experimental Design

This study was carried out on thirty-three male albino mice (22–26 g) body weight. They were supplied by the Animal House Laboratory, Faculty of Pharmacy, Suez Canal University, Ismailia, Egypt. The housing facility’s mean ambient temperature was 22 °C (range: 18–24 °C), and the mean relative humidity varied between 60–80%. The animals were given free access to a typical rodent dietary diet (PICO LAB^®^ Rodent Diet) and clean water was offered *ad libitum* throughout the whole experimental period, 6 weeks. The study was performed according to the Suez Canal University Research Ethics Committee (REC-58-2022; Date April 2022). The LD_50_ of compound I from the intraperitoneal injection of mice was calculated according to the method of Meier and Theakston [52]. Different doses (D) of compound I (2.5, 5, 10, 20, 40, 80, 160, and 320 mg/Kg b.w.) were injected intraperitoneally into 8 mice and then supervised to record the mortality time for each. The survival time (T) of each treated mouse was noted after injection. The values of D/T vs D were used to plot the regression line. For a total of 7 days, the living animals were watched for signs of postponed death. Accordingly; LD_50_ of compound I was estimated to be 40 mg/Kg body weight.

For induction of solid tumor, one male Swiss albino mouse with intraperitoneal transplantation of 1 × 10^6^ viable tumor cells in 0.2 mL of saline was purchased from the National Cancer Institute, Cairo University, Egypt. Was allowed to commercial standard diet and tap water. A balanced salt solution with a pH of 7.4 was used to create tumor cell suspensions, resulting in a final concentration of 5 × 10^6^ viable cells/mL [53]. The harvested cells were diluted with saline to obtain a concentration of 5 × 10^6^ viable EAC cells per mL. A volume of 0.2 mL saline 1 × 10^6^ EAC was injected, once, subcutaneously between the thighs of the lower limb of fifteen randomized male albino mice. Animals were housed for 9 days until the development of solid tumors (Figure 8). 

Twenty-five mice were divided randomly into five equal groups. After 14 days of treatment, the animals were anesthetized and sacrificed, the blood samples were collected by cardiac puncture to be ready for further assays.

#### 3.6.2. Hematological, Biochemical, and Histopathological Assessments

The Abbott CELL-DYN^®^ 1800 automated hematology analyzer (USA) was utilized to evaluate the complete blood count (CBC) using the approved commercial kits (Abbott Laboratories, Abbott Park, IL, USA) [54]. Using commercial kits (Instrumentation Laboratory SpA, Inova diagnostics, Milano, Italy), the activities of aspartate aminotransferase (AST) [55], alanine aminotransferase (ALT) [56], and creatinine level [57] were assessed. For histopathological examination, the samples were paraffin-embedded after being 10% formalin-fixed. Sections of 3 m thickness from each block were provided, mounted to glass slides, and stained with hematoxylin and eosin (H&E) using a 40× objective on a Nikon Ts-2R inverted microscope with constant acquisition settings for all photographs. The scale bar is 20 μm for all images [58].

### 3.7. PARP-1 Inhibition and Molecular Docking 

To assess the inhibitory potency of compound I and compound II against target inhibition of PARP-1 (Bioscience, Cat No. 80580, San Diego, CA, USA) was used. The following equation was used to determine the percentage of medicines that inhibit autophosphorylation [59].

$$\mathrm{Percentage}\;\mathrm{inhibition}=100-\left(\;\frac{\mathrm{Control}}{\mathrm{Treatment}}-\mathrm{Control}\right).$$

For research on molecular modeling, Linux-based systems were employed with Auto Dock Vina and Chimera-UCSF. By measuring the sizes of grid boxes surrounding the cocrystallized ligands, binding sites within proteins were found. Maestro was used in this approach to design and improve the structures of both proteins and chemicals. The chemical PARP-1 (PDB = 5DS3) was docked using the AutoDock 4 and AutoDock Vina [60] using protein structures. Utilizing Vina, the protein and ligand structures were enhanced and energetically favored. By considering binding energy and ligand–receptor interactions, binding activities interpreted the results of molecular docking. After that, the visualization was created using Chimaera [5].

### 3.8. Research Ethics Consideration 

The study was performed according to the Suez Canal University Research Ethics Committee (Approved number REC-58-2022; Date April 2022).

### 3.9. Statistical Analysis

All values were expressed as the mean ± SD. Duncan’s test was performed as a post hoc test for multiple comparisons across all groups after the unpaired Student’s *t*-test and One-Way ANOVA tests to compare means using the GraphPad Prism program, version 8. At *p* < 0.05, differences were considered statistically significant. 

## 4. Conclusions

It was concluded that compound I is more promising than compound II as an anti-proliferative from the *in vitro* and *in vivo* results. cell cycle blocker in critical phases (G1/S phase) and activator of programmed cell death via intrinsic and extrinsic apoptotic cascade pathway rendering the compound I to be a promising modality in more specific and effective anti-liver cancer drugs. It is an effective anti-cancer agent by achieving a commitment decrease in the TIR% and tumor mass, as well as hematological and biochemical parameters. It maintained them near normal levels in SEC-bearing mice. The histopathological evaluation for the compound I showed stopped the deterioration of cancer cells and prevented them from spreading. Compounds I was properly docked inside the binding site of the PARP-1 target protein with stable binding energies and interactive binding modes.

## Figures and Tables

**Table 1:** Effect of the evaluated compounds I, II, and 5-FU on the percentage of the cell viability of MCF-7.

Concentration (µM)	100	25	6.30	1.6	0.4	IC_50_ (µM)
Compound I/MCF-7	30.12 ± 2.30 *	38.18 ± 2.25 *	51.41 ± 2.50 *	63.92 ± 3.30 *	73.41 ± 2.90 *	7.43 ± 0.44
Compound II/MCF-7	37 ± 2.39 *	46.37 ± 2.28 *	55.93 ± 2.58 *	68.43 ± 3.28 *	79.44 ± 3.10 *	16.37 ± 0.97
5-FU/MCF-7	33.81 ± 2.26 *	44.01 ± 2.31 *	53.50 ± 2.66 *	62.46 ± 3.36 *	73.46 ± 3.30 *	10.53 ± 0.58

The assessment compounds I, II, and 5-Fluorouracil (5-FU) are used in serial concentrations from 0.4 to 100 µM. Each value represents the mean ± SD of 3 independent experiments. IC_50_ value represents the concentration at which the evaluated compounds exert half of their maximal inhibitory effect on MCF-7 breast cancer cells by using MTT assay. Compound I refers to N-(2-hydrazineyl-2-oxoethyl)-2-(2.4.5-trichlorophenoxy) acetamide, compound II refers to N-(3-hydrazineyl-3-oxopropyl)-2-(2.4.5-trichlorophenoxy) acetamide and 5-FU refers to, the standard drug, 5-fluorouracil. * Significantly different (*p* < 0.05) from the negative control using the One-Way ANOVA test, followed by Dunnett’s as post hoc for multiple comparisons among all groups.

**Table 2:** Effect of the evaluation compounds I, II, and 5-FU on the percentage of the cell viability of HepG2.

Concentration (µM)	100	25	6.30	1.6	0.4	IC_50_ (µM)
Compound I/HepG2	29.78 ± 3.62 *	37.93 ± 2.91 *	50.88 ± 3.10 *	63.11 ± 2.85 *	73.17 ± 3.20 *	1.43 ± 0.31
Compound II/HepG2	36.62 ± 3.25 *	46.21 ± 3.26 *	55.55 ± 3.25 *	68.18 ± 2.93 *	79.42 ± 3.66 *	6.52 ± 0.86
5-FU/HepG2	33.38 ± 2.50 *	44.64 ± 3.32 *	52.83 ± 3.71 *	62.25 ± 2.90 *	73.11 ± 3.62 *	5.32 ± 0.34

The assessment compounds I, II, and 5-Fluorouracil (5-FU) are used in serial concentrations from 0.4 to 100 µM. Each value represents the mean ± SD of 3 independent experiments. IC_50_ value represents the concentration at which the tested compounds exert half of their maximal inhibitory effect on HepG2 liver cancer cells by using MTT assay. * Significantly different (*p* < 0.05) from the negative control using the One-Way of Various (ANOVA) test, followed by Dunnett’s as Post hoc for multiple comparisons among all groups.

**Table 3:** Effect of compound I on the percentage of the cell viability of HepG2 and THLE-2 cell lines.

Concentration (µM)	100	25	6.30	1.6	0.4	IC_50_ (µM)
Compound I/THLE-2	40.29 ± 2.70 *	54.12 ± 2.25 *	64.52 ± 4.10 *	80.04 ± 3.33 *	88.03 ± 4.25 *	36.27 ± 2.03
Compound I/HepG2	29.78 ± 3.62 *	37.93 ± 2.91 *	50.88 ± 3.10 *	63.11 ± 2.85 *	73.17 ± 3.20 *	1.43 ± 0.31

The evaluated compound I is used in serial concentrations from 0.4 to 100 µM. Each value represents the mean ± SD of 3 independent experiments. IC_50_ value represents the concentration at which the tested compounds exert half of their maximal inhibitory effect on THLE-2 liver normal cells by using MTT assay. * Significantly different (*p* < 0.05) from the negative control using the One-Way ANOVA test, followed by Dunnett’s test as Post hoc for multiple comparisons among all groups.

**Table 4:** Effect of compound I (LD_50_ = 40 mg/Kg B.W) and 5-Fluorouracil on body weight of normal control and SEC-bearing as well as tumor weight and volume on SEC-bearing of mice.

	Groups		Negative Control	Normal + Comp. I	Positive Control (SEC)	SEC + 5-FU	SEC + Comp. I
Parameters							
Mice’s body weight. (g)		1st day	21.85 ± 0.45	22.34 ± 0.20	23.35 ± 0.24	23.90 ± 0.22	24.20 ± 0.46
		15th day	24.25 ± 0.49	25.34 ± 0.22	32.93 ± 0.58	29.97 ± 0.28 *	30.02 ± 0.41 *
Tumor weight (mg)			-----	---	6770 ± 0.20	4320 ± 0.22 *	3890 ± 0.46 *^,#^
Tumor volume (mm^3^)			-----	---	4960 ± 0.30	1760 ± 0.36 *	1260 ± 0.11 *^,#^

Each value represents the mean ± SD, (n = 5). * Significantly different from the SEC control group and ^#^ Significantly different from SEC-bearing mice treated with 5-FU using an unpaired *t*-test (*p* < 0.05).

**Table 5:** Various studied groups of SEC-bearing and normal mice’s hematological, hepatic, and kidney parameters.

Parameters Treatments	Hematological Parameters					Liver Parameters		Kidney Parameters
	RBCs (×10^6^/µL)	Hb (g/dl)	WBC (×10^3^/μL)	Lymphocyte (×10^3^/μL)	Granulocyte (×10^3^/μL)	ALT (U/L)	AST (U/L)	Creatinine (mg/dL)
Negative control	8.33 ± 0.28	13.20 ± 0.61	5.26 ± 0.30	2.20 ± 0.17	2.8 ± 0.2	40 ± 2.10	71 ± 4.04	0.58 ± 0.05
Normal + comp. I	7.97 ± 0.23	13.30 ± 0.28	5.73 ± 0.50	1.88 ± 0.27	3.46 ± 0.41	43 ± 2.88	77 ± 2.10	0.61 ± 0.07
Positive control (SEC)	5.97 ± 0.17	10.03 ± 0.74	10.86 ± 0.73	5.40 ± 0.52	4.26 ± 0.20	62 ± 2.64	139 ± 3.60	0.86 ± 0.15
SEC + comp. I	6.66 ± 0.08 *	12.76 ± 0.75 *	9.90 ± 0.45	4.20 ± 0.10 *	3.50 ± 0.10 *	47.66 ± 2.51 *	111 ± 2.64 *^,#^	0.85 ± 0.10
SEC + 5-FU	6.90 ± 0.36 *	12.45 ± 0.58 *	9.80 ± 0.43	4.34 ± 0.15 *	3.7 ± 0.26 *	53.33 ± 2.88 *	98 ± 4.10 *	0.81 ± 0.06

Each value represents the mean ± SE (n = 5). red Blood Cells (RBCs), Hemoglobin (Hb), white blood cells (WBCs), alanine aminotransferase (ALT), aspartate aminotransferase (AST), and 5-Fluorouracil (5-FU). The data were statistically analyzed by using an unpaired *t*-test; * Significantly different from the SEC positive control group (*p* < 0.05); ^#^ Significantly different from SEC-bearing mice treated with 5-FU (*p* < 0.05).

**Table 6:** The primers used in the current study.

Gene	Forward Primer (5′-3′)	Reverse Primer (3′-5′)
*Bax*	ATGGACGGGTCCGGGGAG	ATCCAGCCCAACAGCCGC
*Bcl2*	AAG CCG GCG ACGACT TCT	GGT GCC GGT TCA GGTACT CA
*p53*	ATGTTTTGCCAACTGGCCAAG	TGAGCAGCGCTCATGGTG
*Casp3*	TGTTTGTGTGCTTCTGAGCC	CACGCCATGTCATCATCAAC
*Casp8*	AGAAGAGGGTCATCCTGGGAGA	TCAGGACTTCCTTCAAGGCTGC
*Casp9*	CATTTCATGGTGGAGGTGAAG	GGGAACTGCAGGTGGCTG

## Data Availability

Upon the request.

## Supplementary Materials

The following supporting information can be downloaded at: https://www.mdpi.com/article/10.3390/ph16111524/s1, Figure S1: Chemical characterization of a phenoxy acetamide derivative (A) represents the ^1^H-NMR analysis spectrogram and (B) represents the ^13^C-NMR analysis spectrogram of compound I.; Figure S2: Chemical characterization of a phenoxy acetamide derivative (A) represents the ^1^H-NMR analysis spectrogram of compound I and (B) represents the ^13^C-NMR analysis spectrogram of compound II.
