# Supplementary material for: In Vitro and In Vivo Effects of Synthesis Novel Phenoxyacetamide Derivatives as Potent Apoptotic Inducer against HepG2 Cells through PARP-1 Inhibition
Source: Pharmaceuticals (Basel). 2023 Oct 26;16(11):1524. doi: 10.3390/ph16111524 (PMC10674780; doi:10.3390/ph16111524)
Supplement: Supplementary file 1 [file pharmaceuticals-16-01524-s001.zip › pharmaceuticals-2678441-supplementary.pdf]

Supplementary data

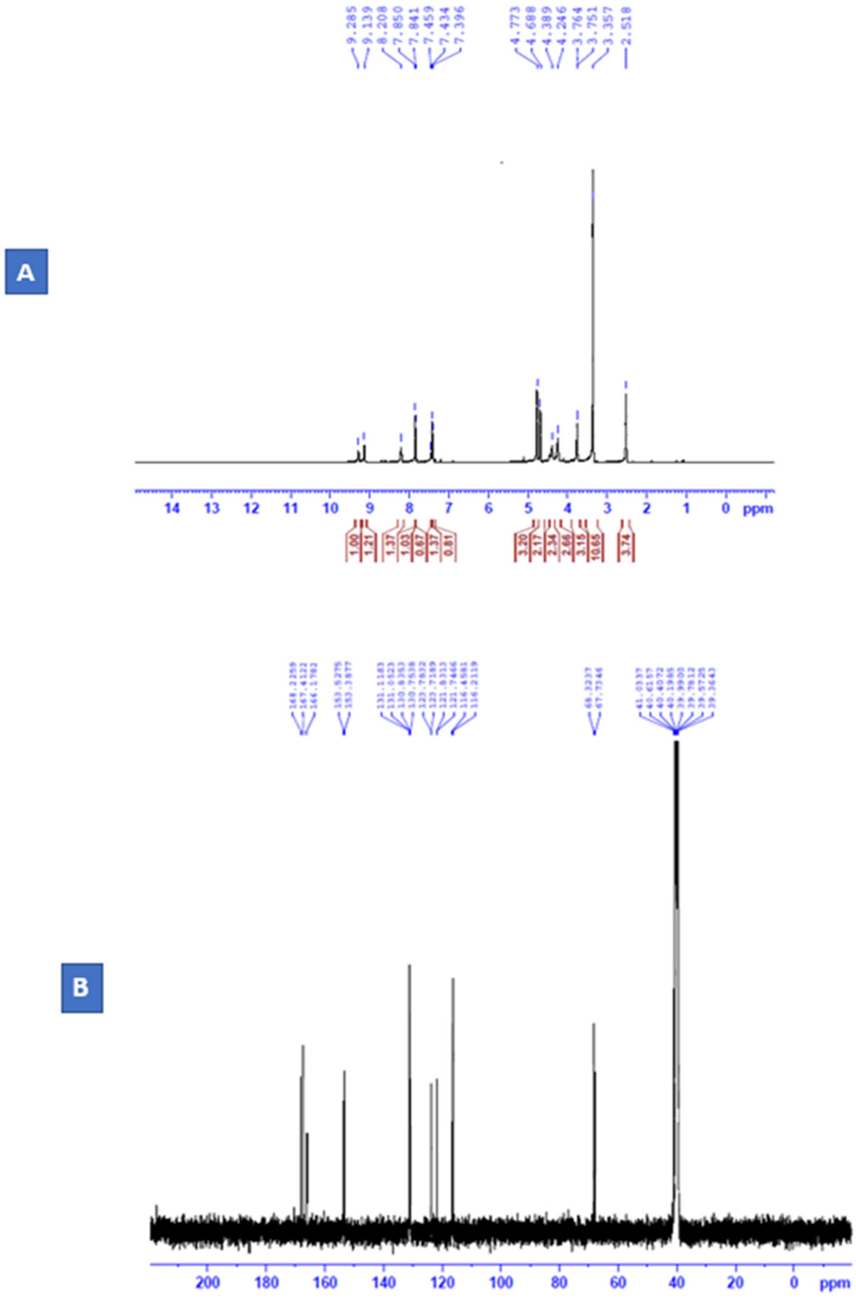

Figure S1. Chemical characterization of a phenoxy acetamide derivative **(A)** represents the ^{1}H-NMR analysis spectrogram and **(B)** represents the ^{13}C-NMR analysis spectrogram of compound I.

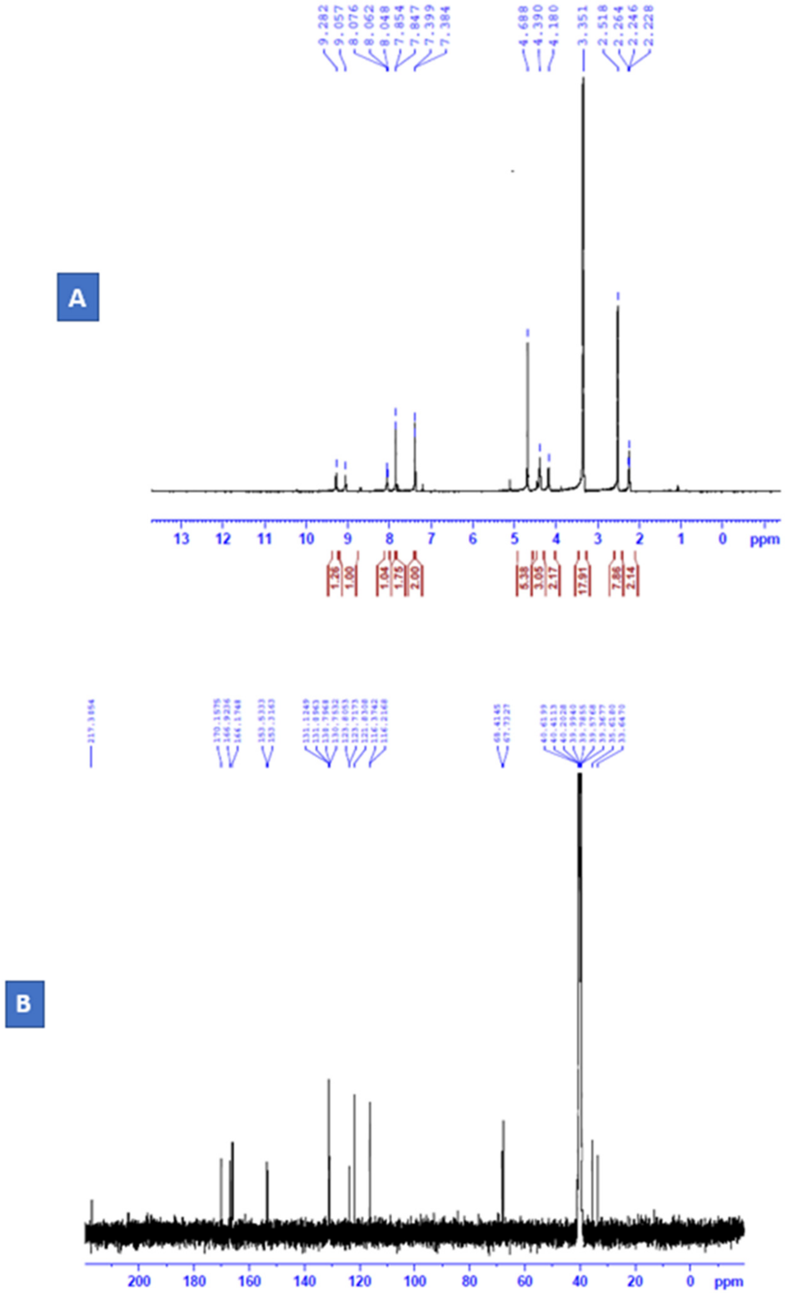

Figure S2. Chemical characterization of a phenoxy acetamide derivative (A) represents the ^{1}H-NMR analysis spectrogram of compound I and (B) represents the ^{13}C-NMR analysis spectrogram of compound II.
